# The expression signature of *in vitro *senescence resembles mouse but not human aging

**DOI:** 10.1186/gb-2005-6-13-r109

**Published:** 2005-12-16

**Authors:** Kristian Wennmalm, Claes Wahlestedt, Ola Larsson

**Affiliations:** 1 Center for Genomics and Bioinformatics, Karolinska Institutet, Berzelius väg 35, 171 77 Stockholm, Sweden; 2 University of Minnesota, Department of Medicine, Minneapolis, MN 55455, USA

## Abstract

**Background:**

The biological mechanisms that underlie aging have not yet been fully identified. Senescence, a phenomenon occurring *in vitro*, limits the number of cell divisions in mammalian cell cultures and has been suggested to contribute to aging.

**Results:**

We investigated whether the changes in gene expression that occur during mammalian aging and induction of cellular senescence are similar. We compared changes of gene expression in seven microarray datasets from aging human, mouse and rat, as well as four microarray datasets from senescent cells of man and mouse. The datasets were publicly available or obtained from other laboratories. Correlation measures were used to establish similarities of the expression profiles and gene ontology analyses to identify functional groups of genes that are co-regulated. Robust similarities were established between aging in different species and tissues, indicating that there is an aging transcriptome. Although some cross-species comparisons displayed high correlation, intra-species similarities were more reliable. Similarly, a senescence transcriptome was demonstrated that is conserved across cell types. A similarity between the expression signatures of cellular senescence and aging could be established in mouse, but not in human.

**Conclusion:**

Our study is the first to use microarray data from several studies and laboratories for dissection of a complex biological phenotype. We demonstrate the presence of a mammalian aging transcriptome, and discuss why similarity between cellular senescence and aging is apparent in aging mice only.

## Abstract 2

A comparison of several microarray datasets from aging human, mouse and rat and datasets from senescent cells from human and mouse shows a similarity between the expression signatures of cellular senescence and aging in mouse but not in humans.

## Background

*In vitro *senescence was discovered as a phenomenon that limits the replicative lifespan of cells grown in culture, and was immediately hypothesised to be a possible cause of human aging [1,2]. During several decades, attempts have been made to establish a link between the age and the replicative potential of cells derived from humans [3]. Although several studies have shown a correlation between *in vivo *and *in vitro *life span, these results have been questioned and re-evaluated [4]. A more convincing approach to establish a link between cellular senescence and aging would be to detect senescent cells *in vivo*. Unfortunately, no good senescence marker exists, except for senescence-associated β-galactosidase (SAβ-gal) staining [5]. This technique has been used extensively but its specificity has been questioned [6]. A correlation between human age and an accumulation of SA-β GAL staining cells has been reported [5] but, for unknown reasons, could not be repeated [4]. In mice, increased SAβ-GAL staining has been reported in animals harbouring the Werner mutation and short telomeres, which gives rise to an aging phenotype [7], following chemotherapeutic treatment [8], in liver regeneration after partial hepatectomy in animals with short telomeres [9] and upon aging [10]. These studies indicate that senescent cells could exist *in vivo *but do not provide any measure of their relative contribution to the aging process.

In the current study, publicly available microarray data describing both aging and *in vitro *senescence were used. By comparing the expression changes that occur during aging in human, mouse and rat across several tissues, we conclude that there is a mammalian aging expression signature. This expression signature is mainly conserved within species, independent of organ, but also highly conserved between some organs between species. Using a similar approach, we also establish that the expression signature of *in vitro *senescence has similarity to that of *in vivo *aging in mouse but not human. This indicates differences between human and mouse aging and suggests that senescence might contribute to aging in mice but not humans.

## Results

### A shared expression profile of aging in mammals

**Figure 1:**
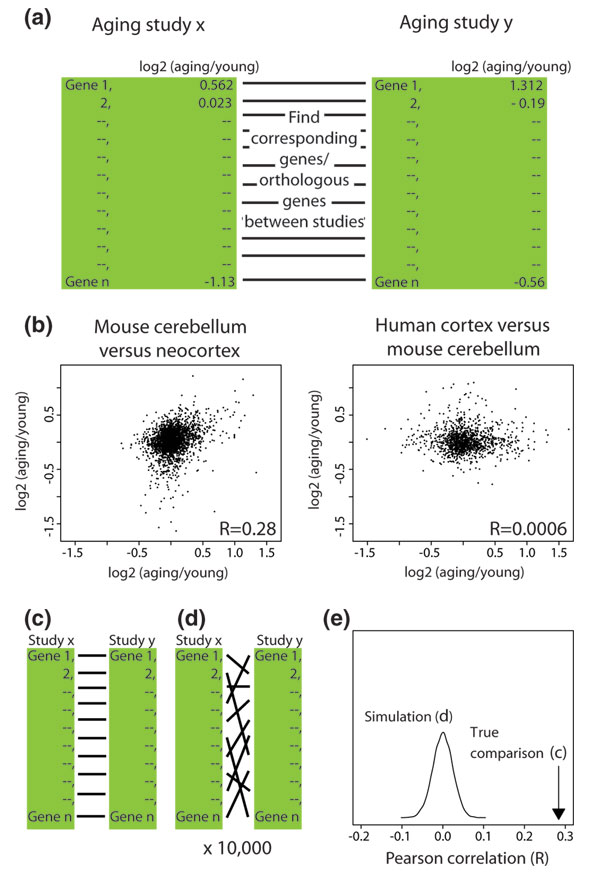
Method for quantifying similarity between gene expression profiles and assessing significance. (**a**) The aging to young ratio of gene expression is calculated and log-transformed for all genes or orthologous genes present on both of the compared microarrays. Ortholog pairs need to be identified in cross-species comparisons. (**b**) Two example scatter plots for comparisons of change in gene expression with age. The calculated correlations (Pearson; R) are displayed to the lower right in the plots. (**c-e**) A distribution of correlations is produced by randomly pairing the genes present on both microarrays 10,000 times and calculating the Pearson correlation for each permutation. The distribution of random correlations (Monte Carlo simulation) is displayed as a density curve in (e). The actual correlation, indicated by an arrow in (e), can then be compared to the random distribution of correlations.

Previous studies have indicated that there is a conserved genetic program for aging across species of low complexity [11]. To date, however, no comprehensive data have indicated that aging in mammals is conserved across species. If a genetic program for aging exists in mammals, it could be conserved across species independent of tissue type, or it could be organ specific. Given the expansion of the microarray field during the past years, resulting in an accumulation of expression datasets in the literature and public databases, we thought that sufficient data were present to enable a study of gene expression signatures during mammalian aging. The multitude of different platforms used to study global expression changes makes such an approach challenging from a data analysis perspective. To make our study possible, we had to control for a possible bias across platforms and species. To achieve this, we calculated a log-transformed ratio of change in gene expression between old and young tissues, for each individual gene in each study. These relative gene expression measures could then be compared to the measures of the same gene, or orthologous gene, in other datasets (Figure 1a). The extent of similarity in a comparison can be visualised using scatter plots (Figure 1b), and a correlation can be calculated to quantify the similarity. It is also necessary, however, to establish what should be considered a significant correlation and what should not. To do this, we used a randomisation approach (Monte Carlo simulation) described in Figure 1c-d. The simulation approximates the likelihood of finding correlation in a comparison if there is no underlying similarity, and can be used to calculate a *P *value for the observed correlation.

**Figure 2:**
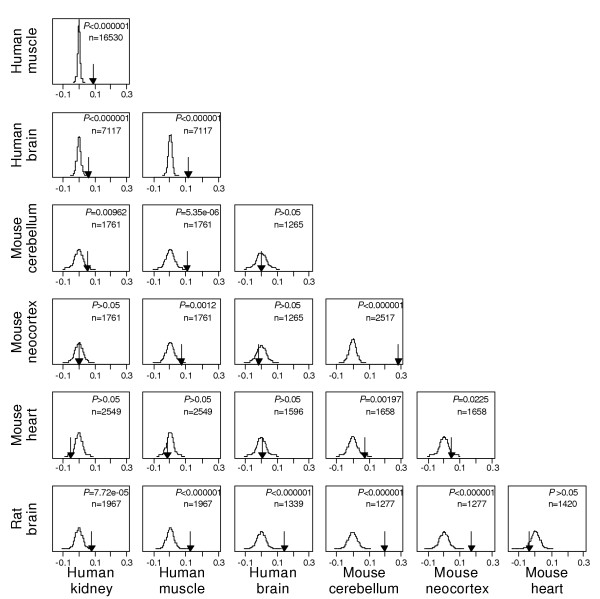
Conserved patterns of gene regulation in mammalian aging. Twenty-one pair wise comparisons of seven aging datasets, with human, mouse and rat data, are displayed. Row and column names indicate the datasets compared. Arrows indicate the Pearson correlation in each comparison. Distributions of correlations produced by random pairing of genes are displayed as density curves. *P *values (calculated using the random correlation distributions as a reference) and numbers of gene or ortholog pairs (present in the individual comparison) are displayed in the top right part of each figure.

**Figure 3:**
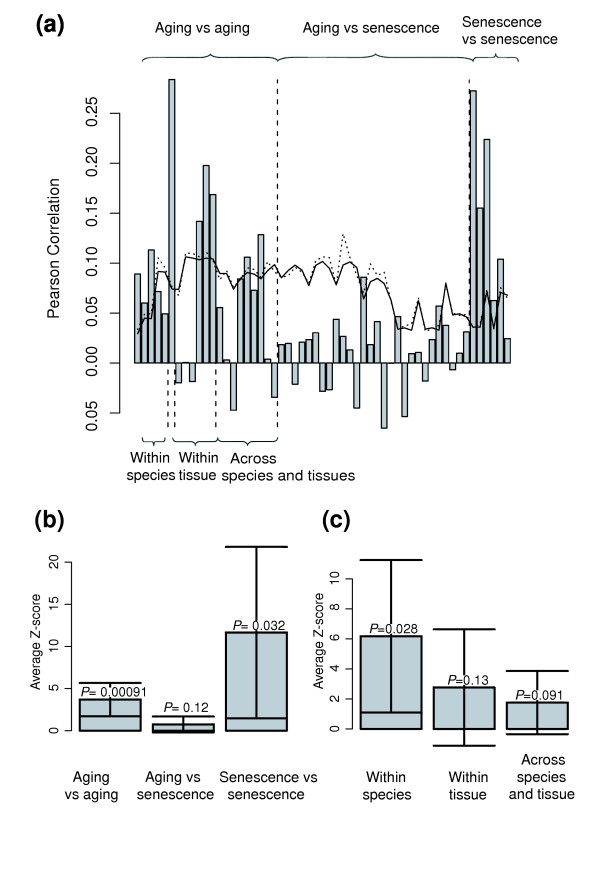
Robust transcriptome signatures of cellular senescence and aging. (**a**) Fifty-five pair wise comparisons of eleven aging and senescence datasets are divided into groups on the basis of the nature of the comparison: aging versus aging (n = 21), aging versus senescence (n = 28), and senescence versus senescence (n = 6). The aging versus aging group is further divided into within species, within tissue, and across species and tissue groups (the highly correlating aging mouse cerebellum to cortex comparison can not be allocated to any of these groups, as it is 'within species and tissue'). Bars represent the correlation in each comparison. The continuous line represents a threshold for significance corresponding to a Bonferroni corrected *P *value < 0.005, as determined by the random distributions of correlations. The dotted line represents the maximum correlation in that distribution (10,000 iterations). (**b**) Analysis of the average Z-score (displacement from the random distribution of correlations in numbers of standard deviations) for the aging versus aging, aging versus senescence, and senescence versus senescence groups. Only the aging versus aging and the senescence versus senescence groups have a mean that passes a *t* test for being different from zero. (**c**) Identical analysis of the within species, within tissue, and across species and tissue groups. Only the within species group has a mean that passes a *t* test for being different from zero. In (b,c), *P *values and tails represent the *P *value for having a mean different from zero, and the 95% confidence interval for the mean, respectively.

A survey of public databases and PubMed identified seven datasets of human, mouse and rat aging that passed technical and quality standards (Materials and methods and Table 1). To look for an aging signature, we compared all seven aging datasets with each other. The density curves of the obtained correlation measures from 10,000 permutations together with the achieved correlation in that comparison are displayed in Figures 2 and 3a (Pearson correlations are displayed throughout this report but similar results were achieved using Spearman and Kendall correlation measures). Of 21 correlations, 10 seemed highly unlikely to occur by chance. A *t *test confirmed that there are shared patterns of gene regulation with age, with a mean correlation of 0.072 (*P *= 0.00039), when the 21 correlations are considered samples from a hypothetical population of age to age transcriptome comparisons (Figure 3b). Negligible similarity was obtained in some comparisons, whereas other comparisons yielded very high correlations. The maximum correlation was obtained when comparing mouse neocortex to mouse cerebellum (0.28) and could be compared to inter-subject variation in gene expression following exercise, with correlations in the same range [12].

3c). The strongest determinant of similarity was species, as this was the only group demonstrating an average Z-score that was larger then zero (*P *= 0.02). Z-scores were preferred in the comparisons as they contain a significance estimate, although similar results were obtained when using correlation measures. The results regarding average correlations within species and tissues indicate a species-specific transcription profile in aging, although some organs show similarities across species (Figure 3c).

We also assessed whether the relationships differed between species and across tissues. We compared the correlations, with regard to their displacement (in standard deviations) from the distribution of simulated correlations, across both species and tissues (Figure 

### Comparing the transcriptomes of *in vitro *senescence and aging

We hypothesised that if senescence is an important mechanism of aging in mammals, then the expression profile of cellular senescence should be detectable in aging tissues. We used the method described above to assess similarities between four senescence datasets (Table 2) and aging (Table 1). As shown in Figure 3a (far right), there is a common transcriptional profile of *in vitro *senescence, as significant similarities were present in five out of six comparisons. The mean correlation of 0.14 (*P *= 0.0077 for having a mean equal or lower than zero) indicates a high degree of similarity across all senescence datasets (Figure 3b). Averages of intra- and inter-species correlations were 0.18 and 0.10, respectively, although it was only possible to compare senescence across cell types within *Homo sapiens*. No average similarity was found in the comparison of *in vitro *senescence and aging (Figure 3a, middle section). Of all 55 possible comparisons of aging and senescence datasets, 19 cases of significant correlation were found, of which 15 were positive and appeared in the aging-aging and senescence-senescence comparison groups. Only four, of which one was negative, appeared in the aging to senescence group, indicating that senescence-like transcription patterns are not generally present in the examined aged tissues of man, mouse and rat. The average deviations from the Monte Carlo simulations (Z-score) in the aging-aging (3.69, *p *= 0.00091), aging-senescence (0.74, *p *= 0.12) and senescence-senescence (11.6, *p *= 0.032) groups show that similarity can not be established in the aging-senescence comparisons (the aging-aging and senescence-senescence comparisons serve as positive controls).

Although these data would suggest that there are no similarities between senescence and aging, we suspected that this might reflect the models used in the senescence studies. In the case of the aging datasets, we compared young and old tissues, where extensive cell division does not take place. In the case of the senescence studies, however, the four senescent transcription profiles were generated by comparisons of logarithmic phase growing cultures to non-growing senescent cultures. We speculated, therefore, that the difference in terms of growth-associated transcriptional changes might obscure the senescence transcriptome signature. To clarify this we studied Gene Ontology categories and demonstrated an overrepresentation of categories related to proliferation in all senescence studies but not in the aging studies (data not shown). To test the hypothesis that cellular senescence is indeed similar to aging (if proliferation is not a component of the comparison), we used two of the datasets where non-growing quiescent cultures had been used to identify senescence specific - not growth-related - gene expression changes. The similarities between the senescent datasets that included a quiescent control were maintained after exchanging the proliferating control for the quiescent control (the correlation was 0.22 when compared to proliferating cells and 0.12 when compared to growth arrested cells).

**Figure 4:**
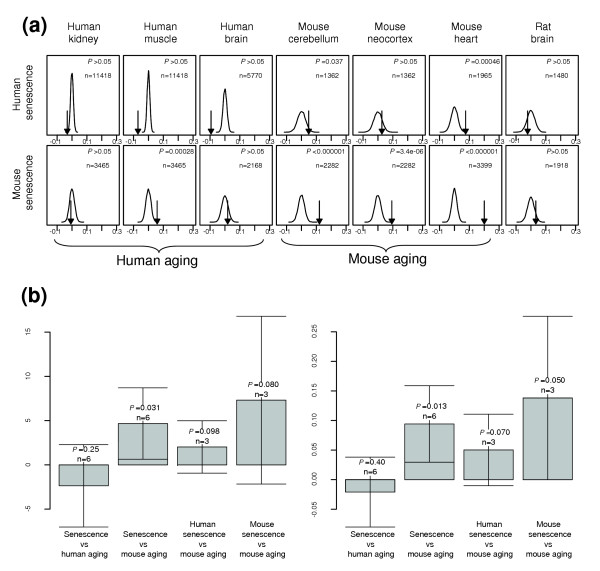
Similarity in the transcriptome signatures of senescence and mouse aging. (**a**) Two senescence datasets, where data derived from proliferating cells (denominator in ratio of differential expression with senescence) have been replaced by data derived from quiescent cells, are compared to seven aging datasets. Arrows represent the Pearson correlation in each comparison. Distributions of correlations produced by random pairing of genes are displayed as density curves. *P *values (calculated using the random correlation distributions as a reference) and numbers of gene or ortholog pairs (present in the individual comparison) are displayed in the top right part of each figure. (**b**) Left: analysis of the average Z-score for groups of comparisons. Only the six comparisons of senescence (mouse and human) and mouse aging (cerebellum, neocortex and heart) have a mean that passes a *t* test for being different from zero. Right: similar analysis of mean Pearson correlations for the same groups, producing the same result. In (**b**), *P *values and tails represent the *P *value for having a mean different from zero, and the 95% confidence interval for the mean, respectively.

Interestingly, when we used the two datasets where senescent cells were compared to quiescent cells and compared these to the aging profiles, we were able to discover a common expression profile between mouse aging and senescence but not human aging and senescence (Figure 4). The average correlation between the senescent datasets and murine aging was 0.094 (*P *= 0.013). To test whether the similarities were significant, we compared the average correlations and Z-scores of groups according to Figure 4. These results show that senescence - or senescence like - patterns of gene expression share similarity with those of mouse *in vivo *aging and further indicate that aging in mouse and human may differ.

To provide additional support for a biological similarity between senescence and mouse aging, we assessed whether the genes that gave rise to this correlation corresponded to biological themes. The contribution of genes associated with a certain Gene Ontology category to the overall correlation in a specific comparison can be assessed. For all six comparisons of senescence and mouse aging, this was done by calculating the partial summation of the Pearson correlation for all appearing Gene Ontology categories. We estimated the probability for chance to produce a correlation between the genes of each individual Gene Ontology category with Monte Carlo simulations. All genes or orthologs in the current comparison were randomly paired and then randomly assigned to Gene Ontology categories. This was repeated 10,000 times to produce distributions of correlations, which were used to calculate *P *values. The results are displayed in Table 3. A conspicuous overlap in Gene Ontology categories between the mouse aging to senescence comparisons was observed. The finding that Gene Ontology categories in many cases overlapped between more than two comparisons indicates that the similarities between senescence and mouse aging correspond to common biological themes. Several of the significant Gene Ontology categories related to mitochondria, protein folding and RNA processing, indicating that these fundamental cellular processes characterise the similarity between mouse aging and cellular senescence.

## Discussion

This is the first microarray meta-analysis where the components of a complex biological phenotype are dissected into its *in vitro *parts. An expression signature was identified that is common to the aging transcriptomes of mouse, man and rat, and conserved mainly within species, but also within organs across species. Evidence of a similarity between the gene expression signatures of aging and cellular senescence was apparent in mouse but, interestingly, not found in human.

The fundamental tool used in this report was estimates of correlations between gene expression changes in aging and senescence. The validity and the accuracy of the significance estimations are, therefore, important. Here, we calculated a ratio of change in gene expression that concurs with the transition from one biological state to another. By comparing changes in gene expression rather than absolute measures several advantages were achieved. Effects on absolute measures of expression level - such as array type differences in ability to quantify transcript abundance, or species and tissue differences in background gene expression - were eliminated. As a consequence, biased correlations within species or between identical arrays are probably of limited concern. Also, all the expression data were used in the comparison, which would not have been the case if genes differentially expressed above a certain threshold had been compared. Thus, the changes in gene expression could be compared to changes in other experiments, regardless of species and the microarray platform used. The ratio approach that we used cannot be applied without considering the biological conditions studied. This is demonstrated by the fact that we were unable to establish similarity between senescence and aging when a gene expression ratio of senescent to proliferating cells was used to describe a senescent transcriptome signature. Similarity became evident when the proliferating cell data were replaced with quiescent cell data, that is, when cell-growth differences no longer obscured the comparison.

Distributions of random correlations were produced for each study-to-study comparison and used to assess the significance of the correlation in that comparison. It might be argued that such simulations underestimate the variance of the true distribution as they assume that all probes (or genes) will display similar changes in signal intensity. In a study [11] where genes were randomly paired within quantiles of overall hybridization intensity, however, the resulting distributions of correlations did not show a significantly increased variance. We consider the statistical methods applied in this study solid, and conclude that many of the correlations obtained in this study are highly unlikely to occur by chance. Yet, it is possible that circumstantial factors may have contributed to unexpected correlations. Any unintentional source of effects on gene expression, transcript abundance or hybridization efficiency that is unevenly distributed between the case and control groups, in two or more datasets, could promote 'false' correlations. The occurrence of such false positive correlations between processes with no known similarity seems to be a lesser issue, however, as a study in which a similar approach to identify similarities of changes in global expression was applied identified no correlations >0.06 when comparing 320 unrelated studies [11]. The correlations of all our summaries that we consider significant were above 0.06. To assess a biological similarity we used several studies and averaged the correlation in the aging-aging, aging-senescence, and senescence-senescence comparison groups. This should further limit the risk of a false positive leading to a false conclusion. A non-significant correlation between processes that indeed are similar would be caused by variation in the individual studies. The main source of variation on the Affymetrix platform, when the normalization approach we used is applied, appears to be biological (compared to technical) [13]. In our approach we carefully assessed the technical quality of the individual studies, thereby limiting the risk of a non-significant result because of technical variation. In addition, each study we used was well replicated and able to show similarities to at least one other study in our comparisons, indicating that the biological variation did not limit the appearance of positive correlations. A full understanding of the risk of false negative correlations would need a large, controlled study where the expression changes of *a priori *known similar processes were compared across, for example, laboratories, platforms and labelling protocols. In our study, the calculated *P *values for the group of expression patterns supported average similarity, indicating that the data should be considered robust. We claim that a valid result can be obtained if the unmodified and robust Monte Carlo simulation is used to approximate significance and the similarities obtained are present in repeated comparisons of similar biological states.

Using these methods, we observed a shared expression signature in mammalian aging. Furthermore, we found that aging is more different across species than across organs of the same species. This finding is intuitive, given the differences between the organisms in terms of life span. Humans have a lifespan that is about 40 times that of mice. It seems possible that the aging phenotype that is allowed to develop during an 80 year life span would be different from that produced during a two to three year life span. In this perspective, it might even seem surprising that some of the cross species but within tissue comparisons give rise to significant correlations. Our findings argue for complexity in the aging program: one component is species specific and is manifested in all organs; another component is organ specific and can give rise to correlation within organs, across species.

An interesting finding in the present study is the detection of a senescence-like component within the murine aging gene expression pattern. This correlation could either occur as a consequence of an accumulation of senescent cells or by a phenotypic change of normal cells towards a senescent phenotype. In either case, the tissue seems to become more similar to senescent cells as it ages. It may appear contradictory that mice, with a much shorter life span and significantly longer telomeres than humans, would display a senescent like phenotype, as senescence is believed to occur as a result of extended cell division and telomere erosion. Cellular stress is an alternative mechanism of senescence induction, however, and the overlap between aging and senescence that we observe could be caused by stress-induced senescence. There are indications in the literature that stress is a general pathway towards senescence; for example, human primary cells can senesce because of replicative exhaustion or cellular stress [14]. A contribution of stress is also observed during RAS induced senescence. Over-expression of RAS seems to induce senescence in cells with high levels of p16 by further increasing the p16 expression. In contrast, cells with low levels of p16 only attain moderate levels of p16 after RAS over-expression and, therefore, do not senesce [15]. Thus, senescence can be induced by both telomeres, via p53/p21, and cellular stress independent of telomere length, characterised by p16 over-expression. The signalling pathways of telomere induced senescence and stress induced senescence are believed to overlap. This is supported by our study where the transcriptional changes with senescence induced by telomere shortening and stress correlate highly. Furthermore, if stress-induced senescence occurs *in vivo *in mice, one would expect p16 to accumulate with age. This is supported by a recent study where p16 was identified as a biomarker of aging in mice in a range of organs [10]. Taken together, these results indicate that aging mouse cells could be stressed *in vivo *and that senescence could occur as a consequence of stress.

Intriguingly, we were unable to establish any similarities between cellular senescence and human tissue aging. One might argue that the low availability of human senescence studies where senescent cells could be compared to quiescent cells limits our analysis. This is unlikely, however, as not only the mouse senescence study, but also the human senescence study, showed similarities to mouse aging. This indicates that the similarities between aging and senescence are robust and less dependent on species. When the quiescence controlled senescence studies and human aging studies were compared, only one comparison showed a significant similarity. Given the robustness of the senescence to mouse aging comparisons, and the overlap between mouse aging and human senescence, it seems likely that if senescence were a pronounced phenotype of human aging, it should have been observed with the technique used presently.

A possible explanation for the difference between the correlation between senescence and aging in mouse and humans, respectively, is the difference in sensitivity to reactive oxygen species. Mice produce more reactive oxygen species [16]. Hence, reactive oxygen species may elicit more pronounced damage in mice than in humans. This idea is supported by the fact that human cells can proliferate indefinitely if telomerase is expressed [17] whereas mouse cells with telomerase eventually enter senescence.

Accumulation of reactive oxygen species could, therefore, induce expression of p16, which in turn might be part of the process of aging in mice. In humans the oxidative stress effect could be expected to be less pronounced and may not induce senescence. The contribution of cellular senescence in human aging may, therefore, be restricted to the telomere driven replicative senescence. One could speculate that senescence indeed occurs *in vivo *in humans, but to a much lesser extent compared to mice. The effect of the senescent cells on the overall function of the organ, and thereby their contribution to aging, would then be expected to be less important. Other possibilities are that apoptosis is the end point of telomere erosion in humans, and that senescence down-stream of telomere erosion is an *in vitro *phenomenon, or that senescence does not generally occur in tissues but only affects stem cells when these fail to escape stress induced senescence or are unable to maintain their telomeres. Regardless of the explanation, our data provide indications that the senescent phenotype does not dominate the aging tissues of humans.

## Conclusion

By comparing changes in gene expression, it is possible to identify conditions that share expression profiles. We found a shared expression profile in aging mammals that seems to be more robust within the species tested but also pronounced within some organs across species. By comparing changes of gene expression that occur during *in vitro *senescence to the aging transcriptomes, senescence like changes in gene expression characterise mouse aging but we could not detect such a similarity in human tissue. Our data provide indications that the senescent phenotype does not dominate the aging tissues of humans.

## Materials and methods

### Data collection and selection

PubMed was screened for studies of senescence or aging where microarrays had been used to monitor global changes in gene expression. Only studies with data derived from human, mouse and rat were included. From the 34 publications that were considered, we were able to use data from 10 (excluded studies are listed in Additional data file 1). Studies using microarrays containing less then 6,000 features were excluded, as subsequent cross-array and cross-species comparisons would become less reliable due to relatively few data points. Other studies were excluded beause we were unable to obtain CEL files, robust multi-array average (RMA) normalized data, or dChip normalized data from Affymetrix studies, or raw data from cDNA-chip studies. Three studies were excluded because of insufficient data quality (details on reasons for exclusion of individual studies are listed in Additional data file 1). The data from the selected studies were either downloaded [18,19] or obtained directly from the authors.

In several cases, we excluded samples from individual datasets if the correlations to replicate samples were dramatically different from other within-replicate correlations (details on data selection can be found in Additional data file 2).

### Normalization

A summary of the normalization methods used for each dataset can be found in Additional data file 2. We normalized all datasets but one, for which we obtained dChip normalized data. All Affymetrix data were normalized with RMA, all cDNA data were normalized using loess normalization with default settings in the Bioconductor package LIMMA.

### Allocation to case and control groups

For subsequent analysis, data needed to be divided into aged and young groups and senescent and control groups. For the aging data, this was straightforward, and the groups are summarized in Table 1. Data on senescence were divided into senescent and control groups. The control group consisted of proliferating or quiescent cells. In the datasets by Zhang *et al*. [20,21], samples from senescent and control populations had been hybridized to the same microarray so that the differential expression between senescent and control populations is represented by a single value (the data belong to a single group). In the dataset by Larsson *et al*. [22], a temperature sensitive inhibitor of senescence (SV40 large T antigen) was used. To subtract differential expression caused by the temperature shift, wild-type (with a temperature insensitive SV40 large T antigen) cells were used as a reference, yielding four groups instead of two: cells with or without the temperature sensitive senescence inhibitor, each divided into permissive temperature groups and restrictive temperature groups. We used data for the 0 h and 72 h time points because the other datasets contained samples from senescent cells harvested days after senescence induction. Additional information on specific datasets is available in Additional file 3.

### Annotation

We used the RESOURCERER tool [23] for annotation and cross-referencing of platforms. Individual features in all datasets were assigned unique and unambiguous identifiers, specifying the gene and its orthologs, thus making cross array and species comparisons possible. For the datasets using Affymetrix MU6500 and U74A chips (annotation information not available within the RESOURCERER tool), additional annotation information was downloaded [24] and used for cross-reference to the identifiers given by RESOURCERER (for details see Additional data file 3).

### Calculation of differential gene expression measures

Normalized log_2 _expression measures were used to produce an expression ratio:

Ri = (1/nj × Σ xj) - (1/nk × Σ xk)

where i is a specific feature on a microarray and x represents the log_2_-transformed expression measures for that feature on all chips in the experiment. Values for x belong to the aging (or senescent; j) group, or to the young (or senescence control; k) group. nj and nk indicate the number of chips in the j and k groups. Thus, the geometric mean of the expression measures from the aging (or senescent) samples was divided by the geometric mean of the expression measures from the young (or control) samples, giving rise to a measure of differential expression with aging or senescence. This removes all expression differences produced by irrelevant variables (for example, chip type and species) because these variables will be identical in the two groups. Ratios from different features, probing the same gene, were averaged. This way of calculating a differential expression ratio was applied to all Affymetrix studies and to all cDNA microarray data where a common reference sample was used for competitive hybridization. In the Zhang *et al*. [20,21] studies, senescent and control samples were competitively hybridized to the same chip, thus no individual expression measures for senescent or control groups can be derived. In this case, the average normalized expression measure for an individual feature across arrays in the experiment was considered an equivalent of Ri and only subjected to averaging within the same gene.

In the study by Larsson *et al*. [22], four groups were used for calculating differential expression, and the expression of the ratio was adapted:

Ri = ((1/nj × Σ xj) - (1/nk × Σ xk)) - ((1/nl × Σ xl) - (1/nm × Σ xm))

Where j, k, l and m correspond to the four groups: cells with a temperature sensitive (j, k) or insensitive (l, m) SV40 large T antigen divided into restrictive (j, l) and permissive (k, m) temperature groups.

### Correlation

Having annotated all datasets using one set of identifiers, and transformed all data for a single gene in an individual dataset to a ratio of differential expression, two or more datasets could be assessed with regards to their correlation. We used Pearson, Spearman and Kendall models of correlation for all comparisons, and they produced consistent results. We have chosen to present Pearson correlations only.

### Monte Carlo simulations

To assess the significance of individual correlations, we iteratively (10,000 times) and randomly paired all genes between the two datasets compared, and calculated the Pearson, Spearman and Kendall correlations. Thus, a distribution of random correlations for each comparison was produced, serving as a reference for each correlation measure.

### Calculation of *P *values for correlation measures

The correlation values produced by the Monte Carlo simulations were normally distributed. We used values for means and standard deviations of these distributions to calculate *P *values for the actual correlation in each comparison. We used Student's *t *tests to assess the means of groups of comparisons, with regards to their *P *values and 95% confidence intervals.

### Confluent cells as a reference for senescence

In the Zhang *et al*. [21] and Larsson *et al*. [22] studies, quiescent/confluent - rather than proliferating - cells had also been used to provide alternative controls for the senescent cell models. To remove effects of cell proliferation differences (between control data in the aging and senescence studies) on the gene expression signature of senescence, we used the data derived from quiescent cells. For the Zhang *et al*. dataset, where the senescence and control samples have been hybridized to the same array, we simply used the senescence versus quiescence data. For the Larsson *et al*. dataset, the log_2 _expression measures from the temperature permissive group was replaced by the expression measures from the confluent group, in the k entry of the ratio expression above.

### Analysis of Gene Ontology categories giving rise to correlation between mouse aging and mouse and human senescence

We analysed the six comparisons of senescence (human and mouse) and mouse aging that showed average correlation. For all Gene Ontology categories, with more than two genes present in a specific comparison, we calculated the partial summation of the Pearson Correlation:

rj = Σ (x_j _- μ_x_)(y_j _- μ_y_)/nσ_x_σ_y_

where j is the set of ortholog pairs associated with that Gene Ontology category, μ_x_, μ_y _and σ_x_, σ_y _are the means and standard deviations of expression ratios for all genes or orthologs appearing in the comparison (the entire study to study comparison), and n is the total number of genes. Statistical significance was assessed by performing Monte Carlo simulations for individual Gene Ontology categories. Random pairing of genes was followed by random assignment to gene ontology categories, and repeated 10,000 times. Calculations of *P *values were performed as described above for the calculation of *P *values of correlations between entire datasets.

## Additional data files

The following additional data are available with the online version of this paper. Additional data file 1 lists the excluded studies and the reasons for their exclusion. Additional data file 2 provides details on data selection and the normalization methods used for each data set. Additional data file 3 provides information on how data was allocated to case (aging or senescent) and control (young, proliferating or quiescent) groups as well as on the cross-referencing of microarrays.

## Supplementary Material

Additional data file 1 The excluded studies and the reasons for their exclusion. Click here for file

Additional data file 2 Details on data selection and the normalization methods used for each data set. Click here for file

Additional data file 3 Information on how data was allocated to case (aging or senescent) and control (young, proliferating or quiescent) groups as well as on the cross-referencing of microarrays. Click here for file

## Figures and Tables

**Table 1:** Included aging studies

Study	Chip type	Species	Tissue	Age
Welle *et al*. [26]	HG U133 A+B	Human	Skeletal muscle	Old (n = 16) 66-77 years
				Young (n = 15) 21-24 years
Lu *et al*. [27]	HG U95 Av2	Human	Frontal cortex of brain	Old (n = 11) 73-106 years
				Young (n = 10) 26-42 years
Rodwell *et al*. [28]	HG U133 A+B	Human	Kidney cortex and medulla	Old (n = 28) 72-92 years
				Young (n = 11) 27-45 years
Lee *et al*. [25]	MU 6500	Mouse	Cerebellum and neocortex of brain	Old (n = 2/3*) 30 months
				Young (n = 3) 5 months
Lee *et al*. [29]	MG U74 A	Mouse	Heart	Old (n = 5) 30 months
				Young (n = 5) 5 months
Blalock *et al*. [30]	RG U34 A	Rat	Hippocampus, brain	Old (n = 10) 24 months
				Young (n = 9) 4 months

All studies of aging that were used in the aging to aging, and the aging to senescence comparisons are listed. Platform, species, tissues and grouping details for each study are shown. The Lee *et al*. [25] data was analyzed as two separate datasets, as it contained data from two different tissues - cerebellum and neocortex, and data on the aging CNS of other species was available for comparison.

**Table 2:** Included senescence studies

Study	Chip type	Species	Cell type	Induction of senescence
Zhang *et al*. [21]	Stanford, cDNA	Human	Fibroblasts	Serial passaging
Schwarze *et al*. [31]	Stanford, cDNA	Human	Prostate epithelial cells	Serial passaging
Zhang *et al*. [20]	Stanford, cDNA	Human	Mammary epithelial cells	Serial passaging
Larsson *et al*. [22]	MG U74 Av2	Mouse	Thymus epithelial cells	Heat inactivation of SV40 large T antigen

All studies of senescence that were used in the aging to senescence comparisons are listed. Platform, species, cell type and senescence model are shown.

**Table 3:** Gene Ontology categories correlating in six comparisons of senescence and mouse aging data sets

Gene Ontology category	Human senescence			Mouse senescence		
	
	Aging mouse cerebellum	Aging mouse neocortex	Aging mouse heart	Aging mouse cerebellum	Aging mouse neocortex	Aging mouse heart
ATP biosynthesis GO:0006754	*P *= 0.00648	-	*P *= 0.019	*P *= 0.00262	*P *= 0.0135	*P *= 0.0222
Hydrogen-exporting ATPase activity GO:0008553	*P *= 0.0284	-	*P *= 0.00701	*P *= 0.00293	-	*P *= 0.00394
Pre-mRNA splicing factor activity GO:0008248	-	-	*P *= 0.0229	*P *= 0.00199	*P *= 0.00533	*P *= 0.000848
Hydrogen ion transporter activity GO:0015078	*P *= 0.0134	-	*P *= 0.00532	*P *= 0.000814	-	*P *= 0.023
Mitotic sister chromatid segregation GO:0000070	-	-	-	*P *= 0.000947	*P *= 0.0462	*P *= 0.0103
Nuclear mRNA splicing via spliceosome GO:0000398	-	-	*P *= 0.022	*P *< 0.000001	-	*P *= 0.000918
Spliceosome complex GO:0005681	-	-	-	*P *= 0.000124	*P *= 0.00589	*P *= 0.0199
Lysosome GO:0005764	-	-	*P *= 0.00545	*P *= 0.00641	-	*P *= 0.0449
Protein folding GO:0006457	*P *= 0.0469	-	-	*P *= 3.47e-06	*P *= 1.25e-05	-
Regulation of cell cycle GO:0000074	-	-	-	*P *= 1.74e-05	*P *= 0.00372	*P *= 0.0083
Metalloendopeptidase activity GO:0004222	*P *= 0.0012	*P *= 0.0115	-	-	-	*P *= 0.00277
Nucleus GO:0005634	-	-	-	*P *= 0.000188	*P *= 0.00143	*P *< 0.000001
Chaperonin-containing T-complex GO:0005832	*P *= 0.0447	-	-	*P *< 0.000001	*P *< 0.000001	-
Negative regulation of cell proliferation GO:0008285	-	-	*P *= 0.00615	*P *= 0.00636	-	*P *= 0.00246
Proton transport GO:0015992	-	-	*P *= 0.0126	*P *= 0.00713	-	*P *= 0.0042
Endoplasmic reticulum GO:0005783	-	*P *= 0.0402	-	-	*P *= 0.0372	*P *= 0.0272
Cytoskeleton GO:0005856	-	-	-	*P *= 0.0347	*P *= 0.0308	*P *= 0.0251
RNA binding GO:0003723	-	-	-	*P *= 0.00305	*P *= 0.0112	*P *= 4.89e-05
Cytoplasm GO:0005737	-	-	-	*P *= 0.0137	*P *= 0.00965	*P *= 5.49e-05
Respiratory gaseous exchange GO:0007585	-	-	-	*P *= 6.25e-05	*P *= 8.77e-06	*P *< 0.000001

Gene Ontology categories correlating in six comparisons of senescence (human and mouse) and mouse aging datasets. Shown are *P *values for partial summations of the Pearson correlation for individual Gene Ontology categories. The *P *values were calculated using Monte Carlo simulations as a reference. Only categories beyond the third branch in the Gene Ontology tree, appearing in more than two of six comparisons, are included. - signifies *p *> 0.05.
