# Supplementary material for: The expression signature of in vitro senescence resembles mouse but not human aging
Source: Genome Biol. 2005 Dec 16;6(13):R109. doi: 10.1186/gb-2005-6-13-r109 (PMC1414108; doi:10.1186/gb-2005-6-13-r109)
Supplement: Additional data file 1 — The excluded studies and the reasons for their exclusion. [file gb-2005-6-13-r109-S1.doc]

| Name | Exclusion criteria | Authors | Journal, Year |
| --- | --- | --- | --- |
| Age-related impairment of the transcriptional responses to oxidative stress in the mouse heart. | 1 | Edwards MG, Prolla TA. | Physiol Genomics. 2003 |
| Comparing genomic expression patterns across species identifies shared transcriptional profile in aging. | 2 | McCarroll SA, Li H. | Nat Genet. 2004 |
| Gene expression profile of aging and its retardation by caloric restriction | 4 | Lee CK,  Prolla TA | Science. 1999 |
| Genomic profiling of short- and long-term caloric restriction effects in the liver of aging mice | 4 | Cao SX, Spindler SR | Proc Natl Acad Sci U S A. 2001 |
| BRCA1 shifts p53-mediated cellular outcomes towards irreversible growth arrest | 4 | Ongusaha PP, Lee SW. | Oncogene. 2003 |
| Mitotic misregulation and human aging | 4 | Ly DH, Schultz PG. | Science. 2000 |
| Aging is associated with increased T-cell chemokine expression in C57BL/6 mice | 4 | Chen J,  Yung RL. | J Gerontol A Biol Sci Med Sci. 2003 Nov;58(11):975-83. |
| T cell chemokine receptor expression in aging | 4 | Mo R,  Yung RL. | J Immunol. 2003 |
| Influences of aging and caloric restriction on the transcriptional profile of skeletal muscle from rhesus monkeys | 4 | Kayo T,  Prolla TA | Proc Natl Acad Sci U S A. 2001 |
| Age-associated changes in gene expression patterns in the liver | 4 | Thomas RP, Evers BM. | J Gastrointest Surg. 2002 |
| Molecular mechanisms of reduced beta-adrenergic signaling in the aged heart as revealed by genomic profiling | 4 | Dobson JG Jr, Pratt RE. | Physiol Genomics. 2003 |
| The effects of aging on gene expression in the hypothalamus and cortex of mice | 4 | Jiang CH,  Hu Y | Proc Natl Acad Sci U S A. 2001 |
| Induction of the Cdk inhibitor p21 by LY83583 inhibits tumor cell proliferation in a p53-independent manner | 4 | Lodygin D, Hermeking H. | J Clin Invest. 2002 |
| Analysis of the effect of aging on the response to hypoxia by cDNA microarray. | 4 | Kim H,  Youn HD. | Mech Ageing Dev. 2003 |
| Influence of ageing, heat shock treatment and in vivo total antioxidant status on gene-expression profile and protein synthesis in human peripheral lymphocytes. | 4 | Visala Rao D, Jones G L | Mech Ageing Dev. 2003 Jan;124(1):55-69. |
| Reproducibility, Sources of Variability, Pooling, and Sample Size: Important Considerations for the Design of High-Density Oligonucleotide Array Experiments. | 4 | Han ES, Hilsenbeck SG. | J Gerontol A Biol Sci Med Sci. 2004 |
| Replicative senescence of activated human hepatic stellate cells is accompanied by a pronounced inflammatory but less fibrogenic phenotype. | 4 | Schnabl B, Brenner D | Hepatology. 2003 Mar;37(3):653-64. |
| Oligonucleotide microarray data mining: search for age-dependent gene expression | 4 | Kirschner M, Radu A | Biochem Biophys Res Commun. 2002 |
| Genome-wide transcript profiles in aging and calorically restricted Drosophila melanogaster | 2 | Pletcher SD, Partridge L. | Curr Biol. 2002 |
| The contributions of sex, genotype and age to transcriptional variance in Drosophila melanogaster | 2 | Jin W,  Gibson G. | Nat Genet. 2001 |
| Effects of dietary folate and aging on gene expression in the colonic mucosa of rats: implications for carcinogenesis. | 3 | Crott JW, Mason JB. | Carcinogenesis. 2004 |
| Molecular determinants of terminal growth arrest induced in tumor cells by a chemotherapeutic agent | 4 | Chang BD, Roninson IB | Proc Natl Acad Sci U S A. 2002 |
| Induction of global stress response in Saccharomyces cerevisiae cells lacking telomerase. | 2 | Teng SC,  Lin JJ. | Biochem Biophys Res Commun. 2002 |
| Transcriptome signature of irreversible senescence in human papillomavirus-positive cervical cancer cells. | 5 | Wells SI, Howley PM | Proc Natl Acad Sci U S A. 2003 |

Supplemental table 1 - excluded studies

1 Anti-correlation with replicate experiments from the same lab, and other aging data

2 Excluded species

3 No differential expression with aging evident when analyzed by SAM

4 Unable to get CEL files, cDNA raw data files, RMA normalized data or dChip normalized data for a number of reasons; authors did not reply, authors distributed files not belonging to any of the listed categories, data was lost, etc

5 Very little positive differential expression evident before correction with temperature-shift controls
