# Supplementary material for: The expression signature of in vitro senescence resembles mouse but not human aging
Source: Genome Biol. 2005 Dec 16;6(13):R109. doi: 10.1186/gb-2005-6-13-r109 (PMC1414108; doi:10.1186/gb-2005-6-13-r109)
Supplement: Additional data file 2 — Details on data selection and the normalization methods used for each data set. [file gb-2005-6-13-r109-S2.doc]

| Study | Data used | Normalization | Selection |
| --- | --- | --- | --- |
| Welle S,  et al. [26] | CEL-files, obtained from authors | RMA | - |
| Lu T, et al. [27] | Excel file with normalized data, at NCBI,  GSE 1572 | dChip | features with negative values removed, reducing data set from 12625 features to 12021 |
| Rodwell GE, et al. [28] | CEL files, obtained from authors | RMA | - |
| Lee CK,  et al. [25] | CEL files, obtained from authors | RMA in R | excluded sample oc4cba, poor correlation to replicates |
| Lee CK,  et al. [29] | CEL files, obtained from authors | RMA | - |
| Blalock EM, et al. [30] | CEL files,  at NCBI,  GSE 854 | RMA | - |
| Zhang H,  et al. [21] | Excel file, at the Stanford Microarray Database (SMD) | loess in R | excluded sample 15996, poor correlation to replicates |
| Schwarze SR, et al. [31] | Excel file,  at SMD | loess in R | excluded three "early senescence" samples, two of a different print type, used 14750,14851,14826 and 14856 |
| Zhang H,  et al. [20] | Excel file,  at SMD | loess in R | used 39906, 40826 and 40828, excluded the others, poor correlation to replicates |
| Larsson O,  et al. [22] | CEL file, obtained from authors | RMA | used late, 72h, time point for senescent samples |

Supplemental table 2a

Data used: the type of data used, and how it was obtained

Normalization: normalization procedures. Robust Multichip Averaging (RMA) was performed with RMAExpress, except in one case where CDF files (for the MU6500 chip) could be obtained for R only, and the RMA normalization was performed in R.

Local Polynomial Regression Fitting (loess) was performed in R.

Selection: In several cases, we excluded samples from individual data sets, if the correlations to replicate samples where dramatically different from other within-replicate correlations.
