# Supplementary material for: The expression signature of in vitro senescence resembles mouse but not human aging
Source: Genome Biol. 2005 Dec 16;6(13):R109. doi: 10.1186/gb-2005-6-13-r109 (PMC1414108; doi:10.1186/gb-2005-6-13-r109)
Supplement: Additional data file 3 — Information on how data was allocated to case (aging or senescent) and control (young, proliferating or quiescent) groups as well as on the cross-referencing of microarrays. [file gb-2005-6-13-r109-S3.doc]

| Study | Groups | Annotation |
| --- | --- | --- |
| Welle S, et al. [26] | men and women pooled | Res.* U133A+B  -> U133 Plus2 |
| Lu T, et al. [27] | men and women pooled | Res.* U95 Av2  -> U133 Plus2 |
| Rodwell GE, et al. [28] | men and women pooled | Res.* U133A+B  -> U133 Plus2 |
| Lee CK, et al. [25] | - | 1) PN600271 murineComp2.xls**  2) Res.*Mu11k  -> U133 plus2 |
| Lee CK, et al. [29] | - | 1) NetAffyx** U74A  -> Locus Link  2) Locus Link  -> Res.* MOE 430v2  -> U133 Plus2 |
| Blalock EM, et al. [30] | - | Res.* RG U34A  -> U133 Plus2 |
| Zhang H, et al. [21] | WS1, WI38, and BJ data pooled, one group*:  senescent vs proliferating (n=7) senescent vs quiescent** (n=5) | Locus Link + UniGene  -> Res.* U133 Plus2 |
| Schwarze SR, et al. [31] | senescent (n=2),  proliferating (n=2) | Locus Link + UniGene  -> Res.* U133 Plus2 |
| Zhang H, et al. [20] | data pooled, one group* | Locus Link + UniGene  -> Res.* U133 Plus2 |
| Larsson O, et al. [22] | four groups,  proliferating:  ts 0h (n=4), ts 72h (n=2),  wt 0h (n=2) and wt 72h (n=2)  quiescent**:  ts 0h replaced by quiescent samples (n=2) | Res.* U74 Av2  -> U133 Plus2 |

# Supplemental table 2b

Groups: information on how data was allocated to case (aging or senescent) and control (young, proliferating or quiescent) groups. For numbers and age intervals in the aging studies (rows one to six), see table 1 of the main article. In two data sets, indicated by *, samples from senescent and control cells had been competitively hybridized, and all data belonged to a single class. Data derived from quiescent cells was available in two data sets (indicated by **). We used this data to calculate an additional ratio, thus removing effects of cell proliferation differences (between control data in the aging and senescence studies) on the gene expression signatures of senescence.

Annotation: detailed information on cross-referencing of microarrays. Res* followed by two names of microarray platforms divided by ->, indicates that a file was downloaded from [http://pga.tigr.org](http://pga.tigr.org/), containing cross references between those microarray platforms. The latter provided the unique and unambiguous identifiers used in this study. For two data sets (indicated by **), using Affymetrix MU6500 and U74A chips, respectively, additional annotation information (a file named PN600271 murineComp2.xls, and several “batch queries” for annotation of the U74A probe ID list) had to be downloaded at [www.affymetrix.com](http://www.affymetrix.com/) and used to cross-reference to identifiers available in cross-reference files at [http://pga.tigr.org](http://pga.tigr.org/).
